# Mice with lymphatic dysfunction develop pathogenic lung tertiary lymphoid organs that model an autoimmune emphysema phenotype of COPD

**DOI:** 10.1152/ajplung.00209.2024

**Published:** 2024-10-22

**Authors:** Barbara Summers, Kihwan Kim, Anjali Trivedi, Tyler M. Lu, Sean Houghton, Jade Palmer-Johnson, Joselyn Rojas-Quintero, Juan Cala-Garcia, Tania Pannellini, Francesca Polverino, Raphaël Lis, Hasina Outtz Reed

**Affiliations:** ^1^Division of Pulmonary and Critical Care, Department of Medicine, Weill Cornell Medicine, New York, New York, United States; ^2^Division of Regenerative Medicine, Department of Medicine, Ansary Stem Cell Institute, Weill Cornell Medicine, New York, New York, United States; ^3^Ronald O. Perelman and Claudia Cohen Center for Reproductive Medicine, Weill Cornell Medicine, New York, New York, United States; ^4^Molecular and Cellular Biology Program, SUNY Downstate School of Graduate Studies, Brooklyn, New York, United States; ^5^Department of Medicine, Section of Pulmonary, Critical Care and Sleep Medicine, Baylor College of Medicine, Houston, Texas, United States; ^6^Department of Pathology, Weill Cornell Medicine, New York, New York, United States; ^7^Department of Cell and Developmental Biology, Weill Cornell Medicine, New York, New York, United States

**Keywords:** autoimmunity, COPD, emphysema, lymphatic vasculature, tertiary lymphoid organs

## Abstract

We have previously shown that mice with a loss of C-type lectin-like type II (CLEC2), which have lymphatic dysfunction due to the role of CLEC2 in platelets for maintaining separation between the venous and lymphatic system, develop lung tertiary lymphoid organ (TLO) formation and lung injury that resembles an emphysema phenotype of chronic obstructive pulmonary disease (COPD). We now sought to investigate whether and how TLOs in these mice may play a pathogenic role in lung injury that is relevant to human disease. We found that inhibiting TLO formation using an anti-CD20 antibody in CLEC2-deficient mice partially blocked the development of emphysema. TLOs in CLEC2-deficient mice were rich in plasma cells and were a source of a broad array of autoantibodies. Chronic cigarette smoke exposure increased the size and number of lung TLOs in CLEC2-deficient mice and was associated with increased markers of antigen presentation and maturation, leading to increased autoantibody deposition. Using lung tissue from patients with COPD, we found an increase in lymphatic markers in patients with an emphysema phenotype and autoreactive TLOs compared with patients with COPD without emphysema that lack prominent TLOs. Taken together, these results demonstrate that emphysema in mice with lymphatic dysfunction can be partially rescued by blocking TLO formation and that these TLOs are the source of autoantibodies that are exacerbated by cigarette smoke. Our work suggests that lymphatic dysfunction in mice may recapitulate some aspects of an autoimmune emphysema phenotype that is seen in a subset of patients with COPD.

**NEW & NOTEWORTHY** The lymphatic vasculature has been implicated in the pathogenesis of lung disease but remains understudied. Here, the authors use a mouse model to show that lymphatic dysfunction leads to a phenotype of emphysema that is characterized by lung tertiary lymphoid organs that are autoreactive and pathogenic. Analysis of human tissue showed increased lymphatic markers in autoimmune emphysema with prominent TLOs, compared with other COPD phenotypes.

## Graphical abstract

**Figure:**
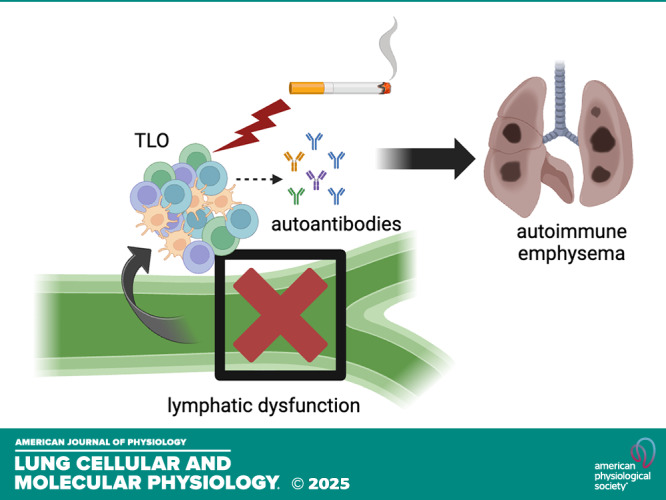

## INTRODUCTION

Tertiary lymphoid organs (TLOs) are a prominent histological finding in diverse lung pathologies, including autoimmune disease, infection, and chronic obstructive pulmonary disease (COPD) (1). These structures resemble secondary lymph nodes in their structure but play context-dependent roles in lung disease. In some settings, TLOs play a protective role by providing local B and T-cell responses, resulting in rapid and sustained immune responses to pathogens. In other settings, TLOs play a pathogenic role, as chronic stimulation of these structures can lead to self-reactive T-cells and B cells that drive autoimmunity. The signals that drive formation of these TLOs may overlap, despite their widely varying functions (1).

The lymphatic vasculature mediates both leukocyte trafficking and antigen drainage in the lungs and plays a key role in lung homeostasis and responses to lung injury (2). Smaller initial lymphatic capillaries in the lung drain into larger collecting lymphatic vessels that are present in the bronchovascular bundles and interlobular septa. These collecting lymphatics drain to the thoracic lymph nodes and eventually into the thoracic duct, where lymph is returned to the blood circulation. Thus, the lymphatic vasculature is uniquely positioned to regulate lung inflammatory responses because of their function in draining fluid and trafficking immune cells in lymph. Changes in lymphatic function have been observed in many settings of lung disease and injury (2). Lymphatics are also found near lung TLOs when they form (3).

We have previously published that impaired lung lymphatic drainage in mice is sufficient to induce TLO formation in the lungs (4). Furthermore, chronic lymphatic dysfunction and TLO formation culminated in lung injury with hallmarks of human COPD, including emphysema (4). We have also found evidence of lymphatic dysfunction in human emphysema and in mice after cigarette smoke (CS) exposure (5, 6). These data and the temporal relationship between lymphatic dysfunction, TLO formation, and emphysema in mouse models suggest a potential role for TLOs formed due to lymphatic impairment in the pathogenesis of CS-induced emphysema, but this has not been previously explored. In this article, we sought to investigate whether TLOs formed due to lymphatic dysfunction play a causal role in emphysema. We found that B cell depletion using an anti-CD20 antibody blocked formation of TLOs and decreased emphysema in mice with lymphatic dysfunction. Lung TLOs formed in mice with lymphatic dysfunction were a source of a broad array of autoantibodies, which had increased lung localization after chronic CS exposure. Chronic CS exposure in mice with lymphatic dysfunction was associated with enlarged lung TLOs and increased markers of maturation in these structures. Using lung tissue from patients with COPD, we found an increase in lymphatic markers in patients with an emphysema phenotype characterized by autoreactive TLOs compared with patients with COPD without emphysema that lack prominent TLOs. Taken together, these results demonstrate that lymphatic dysfunction is associated with pathogenic and autoreactive lung TLOs, which may recapitulate what is seen in an autoimmune emphysema phenotype of COPD.

## MATERIALS AND METHODS

### Mice

*Clec2*^−/−^, *Clec2^flox^*, PF4Cre, and *Prox1-EGFP* (7) mice have been previously described (4, 8) and were maintained on a C57BL/6 background. Mice were housed in the Weill Cornell animal facility in 12/12-h light/dark cycles with ad libitum access to water and food. For all experiments, control and experimental animals were identically housed on the same rack in the animal facility. Littermates or age-matched *Clec2^+/^*^−^ mice or *Clec2^flox^* mice lacking PF4Cre were used as controls for all experiments. Both male and female mice were used in experimental and control groups for all experiments. “*n*” in figure legends indicates biological replicates.

### Lymphatic Endothelial Cell Isolation for Flow Cytometry and Single-Cell RNA Sequencing

To isolate lung lymphatic endothelial cell (LECs), mice carrying the *Prox1-EGFP* reporter [in which all LECs are labeled with green fluorescent protein (GFP)] were injected with Alexa Fluor 647-conjugated isolectin (Invitrogen) for intravital labeling of endothelial cells just before euthanasia, as previously described (9, 10). The lungs were then digested using dispase/collagenase in HBSS to generate a single-cell suspension for FACS, with additional staining using antibodies for CD31 (BioLegend 102418), lineage cocktail (BioLegend 133311), and Epcam (BioLegend 118225). LECs were sorted using positive selection for isolectin, CD31, GFP, and negative gating for Epcam and Lineage markers. For some studies, antibodies for mouse major histocompatibility complex class II (MHC II) (eBioscience 2450719) were also used for flow cytometry.

For single-cell sequencing (PROX1^+^, Pecam1^+^, Ptprc^−^, Lin^−^), LECs from *Clec2^pltKO^* (*n* = 3) and control (*n* = 3) mice were FACS-isolated and sequenced on Illumina’s next-generation sequencing platform. Sequencing reads in FASTQ format were aligned, and filtered feature-barcode matrices were generated for each sample using Cell Ranger (v.6.1.2). Using Seurat (11), cells were retained if they met the following criteria: less than 15% mitochondrial percentage, total counts below 10,000 and between 300 and 5,500 features. This approach was taken to exclude low-quality cells, lysed cells, and doublets. After normalization, 4,000 variable features were selected with the FindVariableFeatures function using the “versus” method. FindNeighbors was then run after scaling using the first 20 principal components and a k.param of 15. Clustering was conducted using Seurat with a resolution of 0.2 and the default settings. Subsequently, UMAP reduction was applied using the first 20 principal components. Contaminating cell types (Ptprc^+^ or PROX1^−^, Cdh5^+^) were then removed, and the remaining cells were renormalized and clustered based on the same parameters. Differential expression was performed using MAST (12) with random effects settings. The MHC II Protein Complex Binding (GO:0023026) Molecular Function gene signature, contributed by the Gene Ontology Consortium, was downloaded from Mouse MySigDb (12). After loading the analysis into Scanpy (13), functional enrichment scores for the signature were calculated for each cell using overrepresentation analysis from decoupleR (14). Tables with top differentially expressed genes and a complete gene list can be found in Supplemental Tables S1 and S4.

### Mouse Model of Cigarette Smoke Exposure and Emphysema

For cigarette smoke exposure studies, we used the inhalation exposure apparatus (TE-10) by Teague Enterprises with 3R4F composition cigarettes (University of Kentucky Center for Tobacco Reference Products). Age-matched mice beginning at 6–8 wk of age were exposed to CS (∼150 mg/m^3^) for a minimum of 3 h per day, 5 days a week for 8 mo (15). Age-matched mice exposed to room air were used as controls. RA control mice were identically housed in the same room, on the same rack, as CS-exposed mice. To assess for emphysema, we calculated mean chord length (MCL). To ensure an accurate representation of the tissue, we used at least eight randomly acquired ×20 images from each mouse, using both the right and left lungs. We used morphometry software to quantify the length of chords within areas identified as airspace (16, 17). Using this method, it is possible to measure the size of the alveoli in all parts of the lung in a standardized and relatively automated manner (16, 17). Large airways, blood vessels, and other nonalveolar structures such as macrophages were manually removed from the images. The experimental group and genotype of the mice were blinded during acquisition and analysis of the images. For B cell depletion, mice were injected with 250 µg of purified rat anti-mouse CD20 (Ultra-LEAF, BioLegend) or rat isotype control antibody retro-orbitally.

### Histology and Immunohistochemistry

Mice were euthanized, and the tissue was perfused with PBS. Before harvest, lungs were inflated with 4% PFA at a constant pressure of 25 cm H_2_O. Lungs were fixed in 4% PFA overnight at 4°. The tissue was then dehydrated and embedded in paraffin for sectioning. Sections (6 µm) were hematoxylin-eosin (H&E)-stained or immunostained with antibodies for B220 (Abcam ab64100), CD3 (Abcam ab5690), CD138 (BioLegend 142502), Fibrinogen (Abcam ab227063), and VEGFR3 (R&D AF743). All primary antibodies were incubated on slides overnight at 4°. After washing, slides were incubated with Alexa Fluor-conjugated secondary antibodies for 1 h at room temperature. Slides were treated with DAPI-containing Vectashield, and a coverslip was applied. Negative control slides were stained with secondary antibodies alone to control for autofluorescence of lung tissue. For the detection of IgG deposition on mouse tissue, an Alexa Fluor-conjugated anti-mouse IgG antibody was used. Western blots were performed according to standard protocols and probed with anti-elastin (Abcam, ab21610) and anti-actin antibodies (Santa Cruz sc-58673). Immunofluorescence was performed either using a Nikon Eclipse microscope (×10 and ×20 objective) with NIS Elements software, an EVOS microscope (×4 objective), or a Zeiss SP8 confocal microscope using Leica software. Image analysis was performed using ImageJ. All images across experimental groups were acquired at the same time for each experiment.

### Autoantibody and Cytokine Assays

Analysis of total IgA, IgG, and anti-collagen I and II in mouse bronchoalveolar lavage (BAL) was done using a commercially available ELISA on a 96-well plate (Chondrex) and a plate reader. Anti-elastin antibodies were analyzed by ELISA made by coating nickel-coated 96-well plates with His-tagged mouse elastin (MyBioSource, MBS2010590). Anti-elastin antibody from Abcam (217356) was used for standards. The plate was blocked with 1% BSA in PBS before incubation with mouse BAL or serial dilutions of anti-elastin standards for 1 h. After washing, horseradish peroxidase (HRP)-conjugated secondary antibodies were added to the plate and incubated for 1 h. The plate was developed using TMB and read at 450/570 nm. An autoantibody array to 128 antigens was performed on mouse BAL or lung lysates (UT Southwestern Genomics and Microarray Core Facility). The samples were treated with DNAse I, diluted, and incubated with an autoantigen array plate. The autoantibodies binding to the antigens on the array were detected with fluorescently labeled anti-IgG and anti-IgA antibodies and scanned with a GenePix 4400A Microarray Scanner. The images were analyzed using GenePix 7.0 software to generate GPR files. The averaged net fluorescent intensity (NFI) of each autoantigen will be normalized to internal controls and was used to generate heat maps. Cytokine profiling of mouse lung homogenates was performed using a mouse proteomic cytokine profiler (R&D Systems) according to the manufacturer’s instructions, which was performed in duplicate.

### Spatial Proteomics

Spatial Proteomics of murine TLOs was performed on FFPE murine lung sections with Nanostring GeoMX for digital spatial profiling (DSP). First, adjacent H&E sections of lung tissue were used to identify TLO structures before region of interest (ROI) selection. Second, slides were treated according to the manufacturer’s instructions. In brief, FFPE sections were deparaffinized, followed by antigen retrieval and probe hybridization with photocleavable oligonucleotide tags. The proprietary proteomic panels were the following: immune cell typing, immune activation status, myeloid, and mouse protein core panels (Supplemental Table S2). Once the slides were scanned, TLO’s were defined as a collection of >40 CD45+ cells (PMID: 36575294). Each ROI sample was collected and used to construct a library for sequencing using NextSeq500 Illumina platforms. Data processing was performed by the Weill Cornell Genomics Core. Proteomic data were audited, normalized, and analyzed using R scripts for GeoMX DSP [“GeomxTools” (10.18129/B9.bioc.GeomxTools), “NanoStringNCTools” (10.18129/B9.bioc.NanoStringNCTools), and “GeoMxWorkflows” (10.18129/B9.bioc.GeoMxWorkflows) packages available from Bioconductor (https://www.bioconductor.org)].

### Spatial Transcriptomics of Human Lung Tissue

For this study, we datamined the transcriptomic data obtained from 48 patients randomly selected for DSP transcriptomic profiling (18). The cohort comprised 8 nonsmoker controls (NSCs), 13 ever-smoker controls, 17 COPD Global Initiative for Chronic Obstructive Lung Disease (GOLD) stage 1-2, and 10 COPD GOLD stage 3-4. Information regarding age, sex, smoking habit status (current vs. former), pack-years, and lung function (FEV1% and FEV/FVC) can be found in Supplemental Table S3. FFPE lung sections from these subjects were treated according to the manufacturer’s instructions. The Chest Imaging Platform software was used by two independent experts to determine the presence of emphysema from computed tomography (CT) chest scans as the percentage ratio of low-attenuation areas below −950 Hounsfield units in each lobe of lung (%LAA-_950_HU). Absence of emphysema was defined as LAA < 5%. FFPE human lung sections were used for Nanostring GeoMX for digital spatial profiling (DSP), according to the manufacturer’s instructions. In brief, FFPE sections were deparaffinized, followed by antigen retrieval, and probe hybridization with photocleavable oligonucleotide tags for whole transcriptome atlas (∼18,000 probes). Parenchyma ROI was defined as Pan-CK+ alveolar epithelial cells. RNA library was sequenced using NextSeq500 Illumina platforms. Data treatment was performed using R scripts for GeoMX DSP [“GeomxTools” (10.18129/B9.bioc.GeomxTools), “NanoStringNCTools” (10.18129/B9.bioc.NanoStringNCTools), and “GeoMxWorkflows” (10.18129/B9.bioc.GeoMxWorkflows) packages available from Bioconductor (https://www.bioconductor.org)]. Significance was determined using mixed linear model set as log2 fold change cutoff ±0.32 and *P* < 0.05.

### Statistics

Data are expressed as means ± SD. Statistical significance was determined by unpaired, two-tailed Student’s *t* test or ANOVA using GraphPad Prism software. *P* values of less than 0.05 were considered statistically significant. Quantification of TLO number in lung tissue was performed using at least five randomly captured ×10 images of H&E-stained lung sections per mouse, representing the entirety of the lung tissue section for each sample. The genotype of the mice was blinded during quantification. TLOs were defined as discrete lymphocyte-dense accumulations on H&E-stained sections. Quantification of the TLO area was performed using H&E sections of the entire lung of each mouse. TLOs were outlined by the free-drawing tool to calculate the TLO area per lung section as a percentage of the total area of lung tissue in ImageJ. Quantification of lymphatic number in lung tissue was performed using at least five randomly captured ×10 images of VEGFR3 staining per mouse. Quantification of microscopic images was performed in a blinded manner.

### Study Approval

All animal experiments were approved by Weill Cornell Medicine Institutional Animal Care and Use Committee.

### Data Availability

RNA sequencing data have been deposited in Gene Expression Omnibus (GEO).

## RESULTS

### B Cell Blockade Prevents TLO Formation and Decreases Emphysema in Mice with Lymphatic Dysfunction

**Figure 1.**
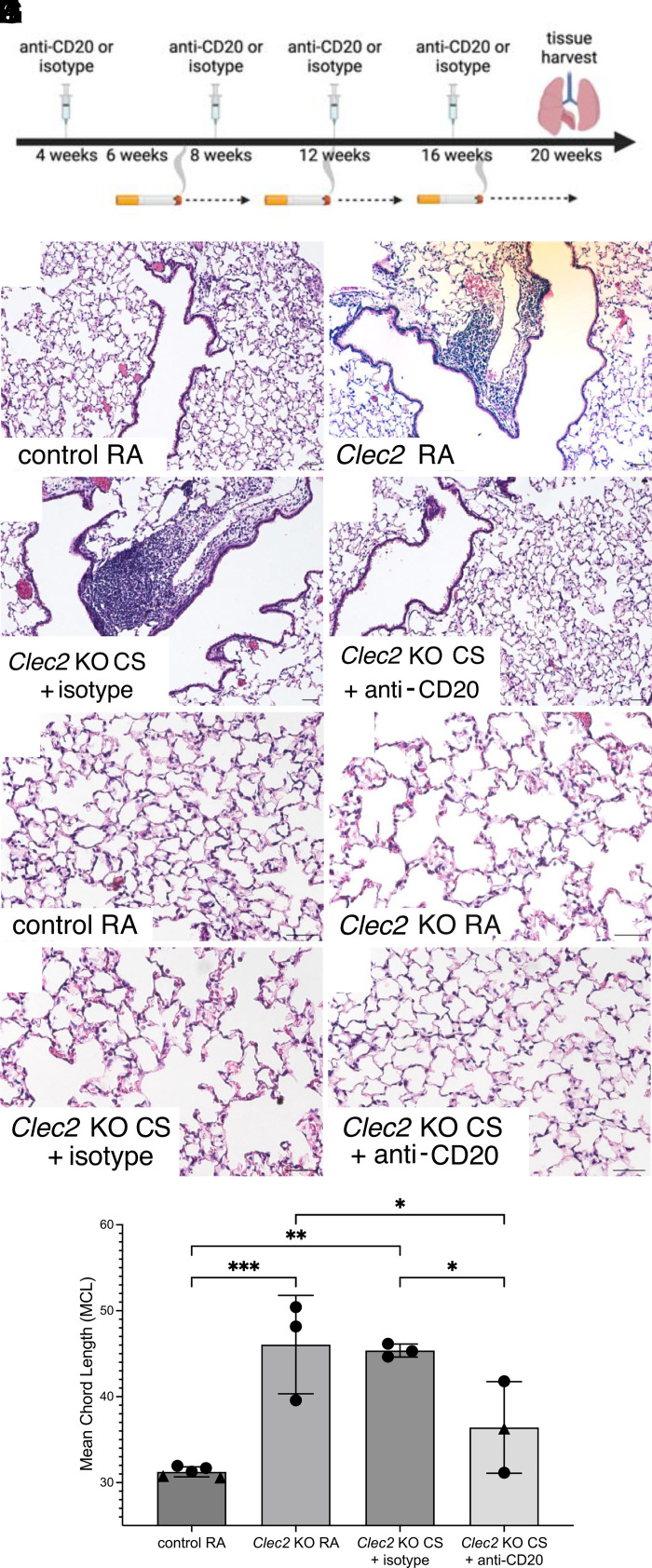
B cell blockade prevents TLO formation and decreases emphysema in mice with lymphatic dysfunction. *A*: experimental design for B cell blockade during chronic CS exposure. *Clec2* KO mice were treated with anti-CD20 or isotype control antibody every 4 wks starting at 4 wks of age. Starting at 6 wk of age, mice were exposed to daily CS exposure for 4 mo before tissue harvest. Image made using BioRender. *B–E*: H&E staining of lung tissue demonstrating TLOs after 4 mo of CS exposure in *Clec2* KO mice exposed to room air (RA) or CS, but not in the lungs of control *Clec2* heterozygous mice or *Clec2* KO treated with anti-CD20 antibody (*B* and *E*). *F–I*: H&E staining of lung tissue after 4 mo of CS or RA in mice treated with anti-CD20 antibody or isotype control. *J*: quantification of alveolar enlargement by mean chord length (MCL). Circles indicate biological replicates of male mice, triangles indicate female mice. Scale bars = 100 µm. All images were acquired at the same time, and quantification was performed in a manner that was blinded to experimental group. All values are means ± SD. *P* value calculated by one-way ANOVA. *P < 0.05, **P = 0.0013, and ***P = 0.0009. CS, cigarette smoke; CLEC2, C-type lectin-like type II; H&E, hematoxylin-eosin; KO, knockout; TLO, tertiary lymphoid organ; RA, room air.

We sought to investigate the role of TLOs formed due to lymphatic dysfunction in the pathogenesis of cigarette smoke (CS)-induced emphysema using a mouse model. As there are currently no genetic models of lung-specific lymphatic dysfunction, we used mice with globally impaired lymphatic drainage that form lung TLOs, which we and others have previously characterized. Mice lacking C-type lectin-like type II (CLEC2), a platelet receptor that is activated by Podoplanin (PDPN) on the lymphatic endothelium, have impaired lymph flow due to the lack of separation between the venous and lymphatic vasculature (19–22). In the absence of CLEC2, there is retrograde flow of blood that creates back pressure from the venous system into the lymphatic system at thoracic duct, impairing lymph flow and resulting in flow-dependent defects in vessel remodeling and valve formation (4, 8, 20, 21). We have shown that chronically impaired lymph flow and TLOs in *Clec2* knockout (*Clec2* KO) mice result in a lung injury that resembles emphysema (4). We now tested the effect of chronic CS exposure in CLEC2 knockout (KO) mice compared with control littermates that were heterozygous for *Clec2* (Fig. 1*A*). We found that TLOs persist, if not enlarge, with chronic CS exposure (Fig. 1, *B*–*D*). B cell blockade with an anti-CD20 antibody was sufficient to significantly inhibit lung TLO formation in these animals, reinforcing the key role of B cells in the organization of these structures (Fig. 1, *A*–*E*). Lymphatics continued to be abnormal in *Clec2* KO mice after TLO inhibition, as evidenced by the persistence of histologically abnormal lymphatic vessels in the lungs of these mice (Supplemental Fig. S1). Interestingly, CS exposure in *Clec2* KO mice did not result in worse emphysema, as quantified by mean chord length (MCL) (Fig. 1*J*), perhaps due to the already severe emphysema seen in CLEC2 KO animals at baseline, as we have previously seen (4). However, loss of TLOs due to anti-CD20 antibody led to decreased emphysema in *Clec2* KO mice (Fig. 1, *F*–*J*), despite continued lymphatic dysfunction in these animals, suggesting a pathogenic role of these TLOs in lung injury.

### Lung TLOs in Mice with Lymphatic Dysfunction Produce Autoantibodies

**Figure 2.**
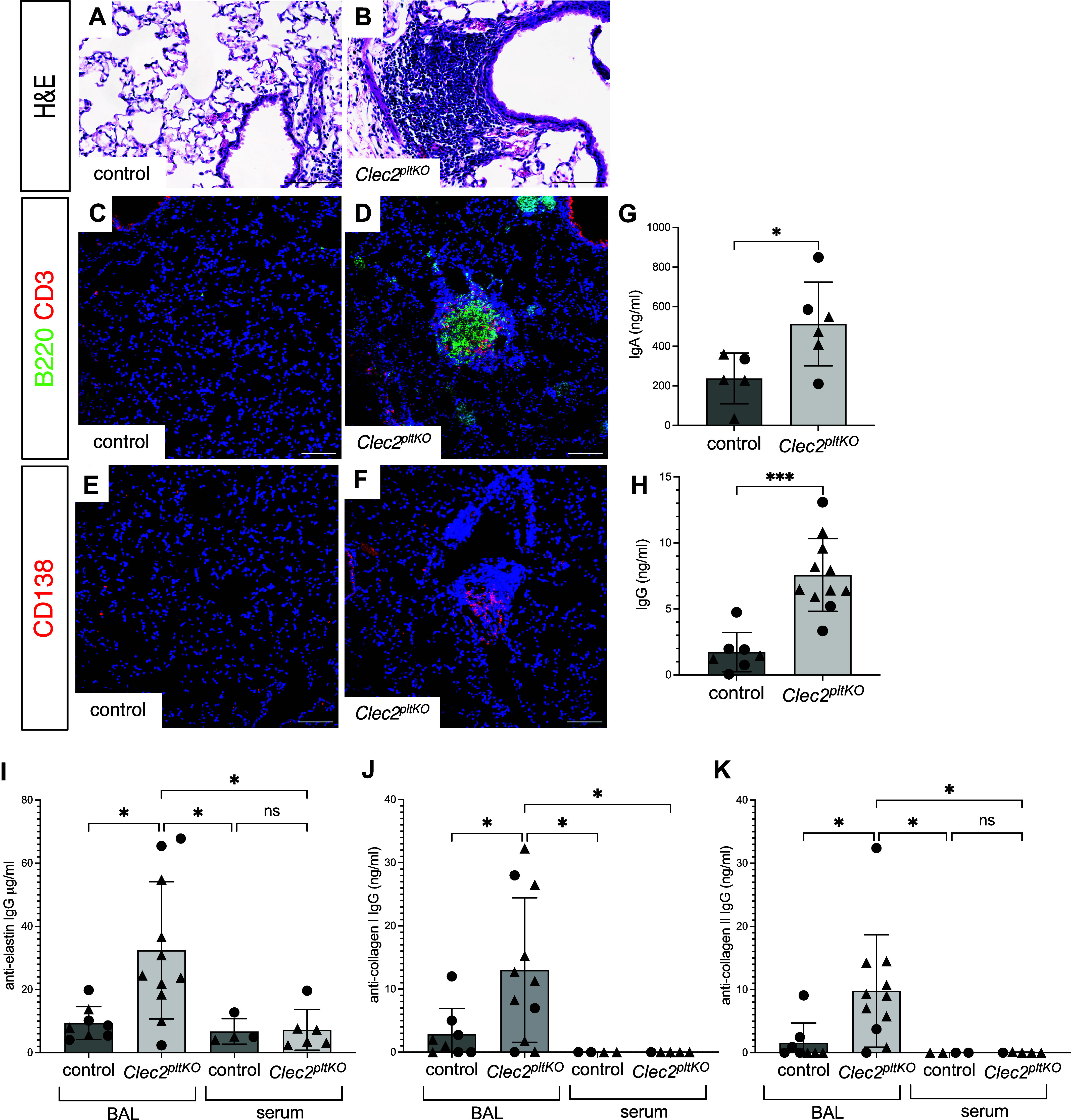
TLOs in mice with lymphatic dysfunction are associated with autoantibodies to extracellular matrix proteins. H&E staining of lung tissue from control *Clec2^flox^* mice lacking PF4Cre (*A*) and *Clec2^pltKO^* (*B*) mice. Immunohistochemical staining of lung tissue from control (*C* and *E*) and *Clec2^pltKO^* (*D* and *F*) mice. Slides were stained with antibodies for B220 and CD3 (*C* and *D*) or CD138 (*E* and *F*) and counterstained with DAPI. *G* and *H*: ELISA for total IgA and IgG in BAL fluid from control and *Clec2^pltKO^* mice. *P* value calculated by Student’s *t* test, *P = 0.0318 in *G*, and ***P = 0.0001 in *H*. *I–K*: ELISA for anti-elastin IgG (*I*), anti-collagen I IgG (*J*), and anti-collagen II IgG (*K*) in BAL fluid and serum from *Clec2^pltKO^* and control mice. All images are representative of at least eight biological replicates from two separate experiments. Scale bars = 100 µm. All values are means ± SD. *P* value in *I–K* calculated by one-way ANOVA. *P < 0.05 for *I–K*, ns = not significant.). Circles indicate biological replicates of male mice; triangles indicate female mice. BAL, bronchoalveolar lavage; H&E, hematoxylin-eosin; TLO, tertiary lymphoid organ.

To further explore the role of TLOs formed due to lymphatic dysfunction on lung pathogenesis and because CLEC2 is expressed on some immune cell types (most notably dendritic cells) and do not have normal lymph node development (23), we generated mice with platelet-specific loss of CLEC2 (*Clec2^fl/fl;^PF4Cre*, hereafter *Clec2^pltKO^*). *Clec2^pltKO^* mice are born at expected frequencies, survive to adulthood, develop lymph nodes, and have nearly identical lung TLOs formation and emphysema development as we have seen with global loss of CLEC2 (Fig. 2, *A* and *B*). TLOs in *Clec2^pltKO^* mice consist predominantly of B cells, including plasma cells (Fig. 2, *C*–*F*), and were not typically seen in the lungs of control *Clec2^flox^* mice that lack PF4Cre-mediated deletion. We found that TLOs in *Clec2^pltKO^* mice are associated with an increase in total IgA and IgG antibodies in the bronchoalveolar lavage (BAL) fluid of these animals (Fig. 2, *G* and *H*). Given the increase in total antibody production in *Clec2^pltKO^* mice, we next assessed whether self-reactive antibodies were also present. Interestingly, we found that autoantibodies to collagen and elastin were significantly increased in the BAL fluid, but not the serum, of *Clec2^pltKO^* mice compared with control mice (Fig. 2, *I*–*K*). Given that loss of CLEC2 is associated with predominantly lung TLOs despite systemic lymphatic dysfunction (4, 8, 20), the lack of serum autoantibodies in these mice suggests the specific role of lung TLOs in autoantibody production in this model.

**Figure 3.**
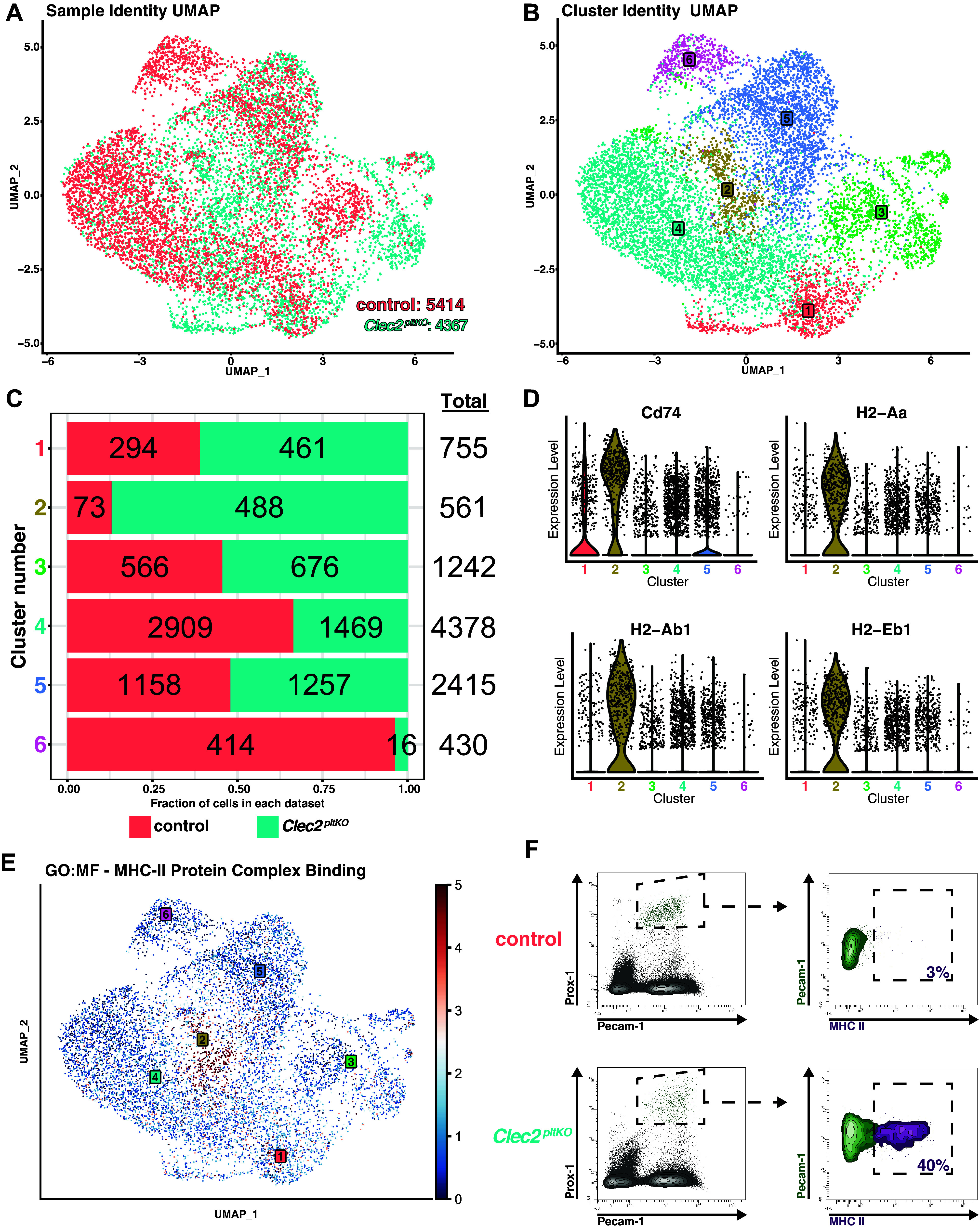
MHC II-expressing LECs in mice with lymphatic dysfunction. *A*: sample identity UMAP plot of control and *Clec2^pltKO^* mouse lung LEC samples from (*n* = 3) biological replicates per group totaling 5,414 control cells and 4,367 *Clec2^pltKO^* cells. *B*: cluster identity UMAP plot demonstrating six distinct cell clusters across all the LECs in our dataset. *C*: cluster composition plot showing the distribution of control *Clec2^flox^* and *Clec2^pltKO^* LECs in each distinct cluster as well as the total cell number per cluster, which highlights cluster 2 as a predominantly *Clec2^pltKO^* cluster. *D*: violin plots demonstrating significant differential expression of select MHC II pathway genes with cluster 2 showing the highest expression across all markers. *E*: pathway enrichment UMAP plot demonstrating the expression of MHC II protein complex binding (GO:0023026) molecular function gene signatures across all cells revealing enrichment in the *Clec2^pltKO^* dominant cluster 2. *F*: flow cytometry plots showing PROX1^+^ Pecam-1^+^ LECs (Ptprc^−^ Lin^−^) pooled from control (*n* = 5) vs. *Clec2^pltKO^* (*n* = 3) mice. LEC, lymphatic endothelial cell; MHC II, major histocompatibility complex class II.

Previous studies have shown an immunomodulatory subtype of lymphatic endothelial cell (LEC) in mice with impaired lymphatic function that form TLOs (24). We performed single-cell RNA sequencing on isolated lung LECs from control and *Clec2^pltKO^* mice and similarly found a LEC subtype characterized by the MHC II antigen presentation gene *CD74* and class II histocompatibility genes *H2-Aa1*, *H2-Ab1*, and *H2-Eb1* in *Clec2^pltKO^* mice (cluster 2) but not control *Clec2^flox^* mice (Fig. 3, *A*–*D*). Pathway analysis also demonstrated enrichment of MHC II protein complex binding in cluster 2 that is overwhelmingly unique to lung LECs from *Clec2^pltKO^* mice (Fig. 3*E*), which was confirmed by flow cytometry (Fig. 3*F*). We also identified an LEC population that was enriched in *Clec2^pltKO^* mice and marked by the expression of *Ptx3*, *Mrc1*, and *Nrp2*, resembling a LEC subtype that has been previously found at terminal lymphatic capillaries and may mediate immune cell recruitment and entry as well as lymphangiogenesis (Supplemental Table S1) (25–27). These results agree with previous studies and suggest that inflammation and lymphatic dysfunction with TLO formation are associated with changes in immunomodulatory LEC populations.

### CS Exposure Leads to Lung TLO Expansion and Increased Markers of Antigen Presentation

**Figure 4.**
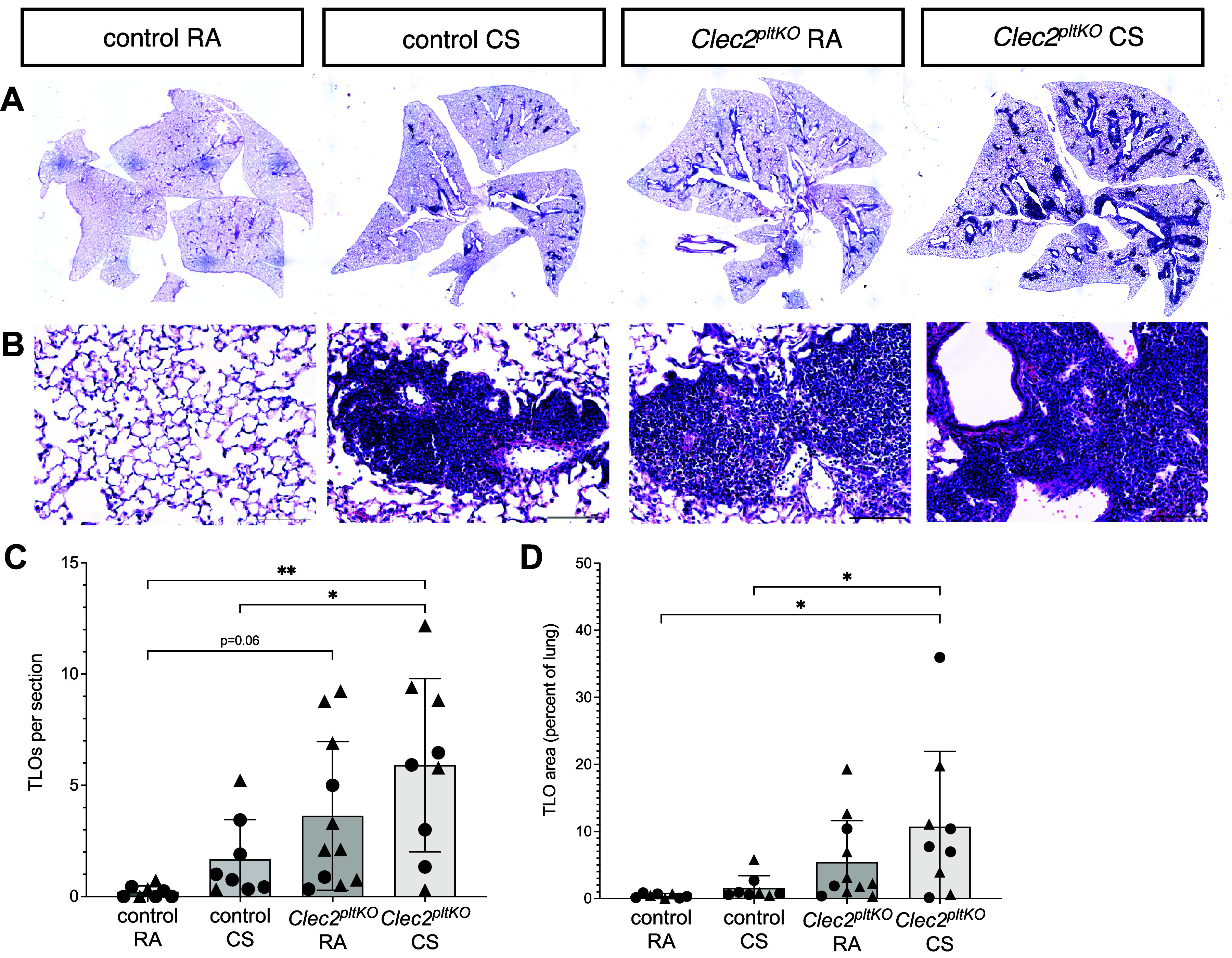
CS exposure leads to lung TLO expansion. *A* and *B*: H&E staining of lung sections from control and *Clec2^pltKO^* mice after 8 mo of exposure to CS or age-matched room air (RA) *Clec2^flox^* controls. Scale bars = 100 µm. All images were acquired at the same time, and quantification was performed in a manner that was blinded to experimental group. Images are representative of biological replicates of *n* = 8 control RA mice, *n* = 8 control CS mice, *n* = 11 *Clec2^pltKO^* RA mice, *n* = 9 *Clec2^pltKO^* CS mice. *C* and *D*: quantification of TLOs number and size per whole lung section in control and *Clec2^pltKO^* mice exposed to 8 mo of CS or RA. All values are means ± SD. *P* values calculated by one-way ANOVA. *P = 0.02 and **P = 0.0013 in *C*, *P < 0.05 in *D*. Circles indicate data points for male mice, triangles indicate female mice. CS, cigarette smoke; H&E, hematoxylin-eosin; TLO, tertiary lymphoid organ.

**Figure 5.**
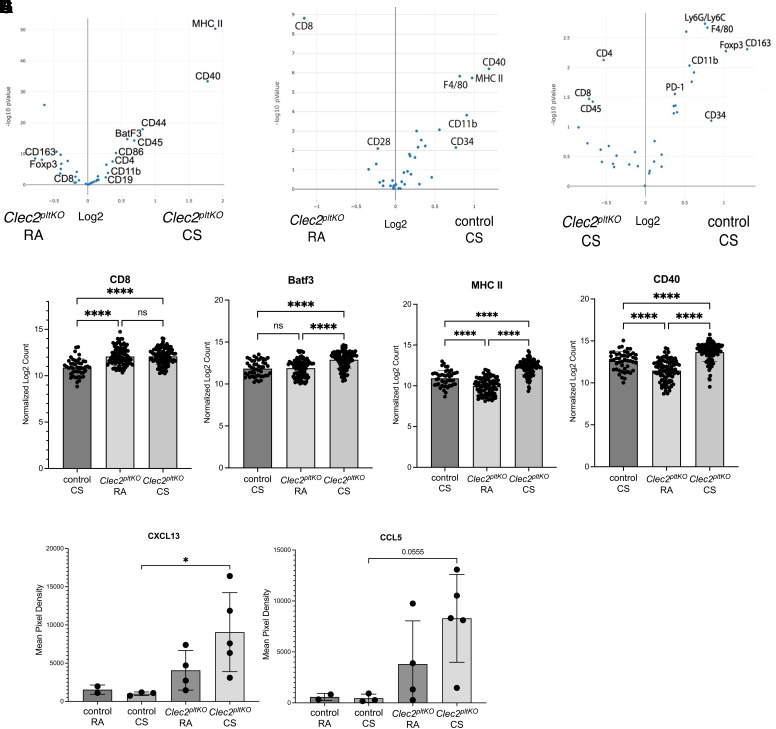
Increased markers of antigen presentation in TLOs after CS exposure. *A–C*: volcano plots showing protein expression of immune cell and activation markers TLOs from *Clec2^pltKO^* mice exposed to 8 mo of RA or CS, as well as control mice exposed to 8 mo of CS. Data were generated by NanoString GeoMX digital spatial profiling with ROI defined as 8–16 TLOs from *n* = 6 control CS, *n* = 7 *Clec2^pltKO^* RA mice, *n* = 7 *Clec2^pltKO^* CS mice. Log2 fold change is on *x*-axis, −log10 *P* value on *y*-axis. *D*–*F*: quantification of CD8 (*D*) Batf3 (*E*) MHC II (*F*), and CD40 (*G*) in TLOs from *Clec2^pltKO^* mice exposed to RA and CS, as well as control mice exposed to CS by GeoMX digital spatial profiling. Each dot represents an ROI corresponding to a distinct TLO. All values are means ± SD. *P* values calculated by one-way ANOVA, ****P < 0.0001. *H* and *I*: ELISA for CXCL13 (*H*) and CCL5 (*I*), using whole lung lysates from mice exposed to CS or RA for 8 mo. All values are means ± SD. *P* values calculated by one-way ANOVA, *P = 0.0478. CS, cigarette smoke; MHC II, major histocompatibility complex class II; RA, room air; ROI, region of interest; TLO, tertiary lymphoid organ.

We next tested the effect of CS on lung TLOs. We found that CS exposure caused an increase in the size and number of lung TLOs after CS exposure (Fig. 4*A*). Although control *Clec2^flox^* mice exposed to chronic CS develop lung TLOs as has been previously reported (28), *Clec2^pltKO^* mice demonstrated expansion of their preexisting TLOs that also had a more dense histologic appearance, though this is not statistically significant (Fig. 4, *B*–*D*). Given this, and to more quantitatively investigate TLO composition and activation after CS exposure, we used GeoMx digital spatial proteomic profiling (NanoString) to compare TLOs formed from CS exposure in control and *Clec2^pltKO^* mice, compared with TLOs in *Clec2^pltKO^* mice exposed to room air. We defined our regions of interest (ROIs) as lung TLOs and used an antibody panel consisting of 28 targets toward both immunophenotyping as well as immune activation (Supplemental Table S2). Interestingly, we detected only small differences in B cell, T cell, and myeloid markers in TLOs from these animals with the exception of significantly decreased CD8^+^ T cells in TLOs from CS-exposed control mice compared with *Clec2^pltKO^* mice (Fig. 5, *A*–*D*). However, we found significantly increased Batf3, MHC II and CD40, proteins involved in antigen presentation, class switching, and memory (29, 30), in *Clec2^pltKO^* mice that were exposed to CS compared with TLOs from room air exposed *Clec2^pltKO^* mice (Fig. 5, *A*–*G*). ELISA on lung samples revealed an increase in CXCL13 and CCL5 cytokines that are associated with the formation and maintenance of TLOs, as well as B cell/T cell cross talk (31–35), in *Clec2^pltKO^* mice exposed to CS compared with control CS-exposed mice (Fig. 5, *H* and *L*). These results suggest that CS promotes both the formation of TLOs as well as the maintenance and activation of preexisting TLOs formed due to lymphatic dysfunction.

### CS Exposure Causes Increased Lung Autoantibody Localization in Mice with Lymphatic Dysfunction

**Figure 6.**
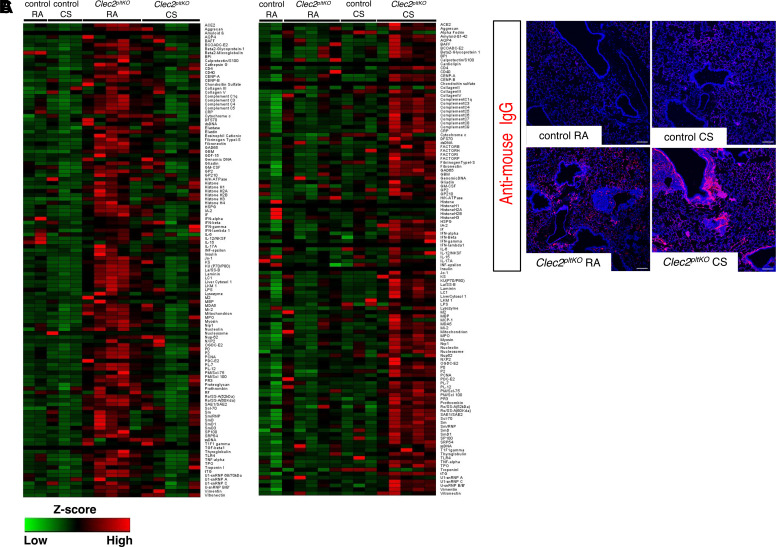
Increased lung autoantibodies localization after CS exposure in mice with lymphatic dysfunction. *A*: heatmap of IgG autoantibodies to 128 antigens detected by ELISA using BAL fluid from *Clec2^pltKO^* and control *Clec2^flox^* mice exposed to 8 mo of CS or RA and fluorescently labeled mouse anti-IgG. *B*: heatmap of IgG autoantibodies to 128 antigens detected by ELISA using lung lysates from *Clec2^pltKO^* and control mice exposed to 8 mo of CS or RA and fluorescently labeled mouse anti-IgG. Fluorescence intensity was normalized to internal controls. ELISAs for *A* and *B* were performed biological replicates of *n* = 2 control *Clec2^flox^* RA mice, *n* = 3 control *Clec2^flox^* CS mice, *n* = 5 *Clec2^pltKO^* RA mice, *n* = 5 *Clec2^pltKO^* CS mice. *C*: detection of IgG deposition in lung tissue from *Clec2^pltKO^* and control mice exposed to 8 mo of CS or RA using fluorescently labeled anti-mouse IgG. Images are representative of biological replicates of *n* = 8 control *Clec2^flox^* RA mice, *n* = 8 control *Clec2^flox^* CS mice, *n* = 11 *Clec2^pltKO^* RA mice, and *n* = 9 *Clec2^pltKO^* CS mice. Scale bars = 100 µm. BAL, bronchoalveolar lavage; CS, cigarette smoke; RA, room air.

Previous studies have shown lymphatic dysfunction and TLO formation in diverse settings of autoimmune and autoinflammatory disease, including rheumatoid arthritis, Chron’s disease, and systemic lupus erythematosus (SLE) (36–40), and that CS exposure causes more severe disease in these patients (41–43). Similarly, CS promotes autoantibody production and disease progression in patients with autoimmune emphysema, a COPD phenotype that is characterized by prominent autoreactive TLOs (18, 43–45). These autoantibodies target a broad array of antigens, including ones that are not specific to the lung and more commonly seen in autoimmune diseases such as SLE and rheumatoid arthritis (46). We quantified autoantibody production after CS exposure using an autoantigen microarray to detect autoantibodies to a broad range of targets. We found that IgG autoantibodies were broadly increased in the bronchoalveolar lavage (BAL) fluid *Clec2^pltKO^* mice compared with control *Clec2^flox^* mice (Fig. 6*A*). Chronic CS exposure dramatically increased autoantibodies within the lung tissue of *Clec2^pltKO^* mice, but not in the BAL, suggesting a shift in their localization (Fig. 6, *A* and *B*). A similar pattern was seen for IgA autoantibodies (Supplementary Fig. S2). Histological analysis confirmed lung localization of autoantibodies after CS exposure in *Clec2^pltKO^* mice, which were present throughout the parenchyma and along airways and arteries (Fig. 6*C*). CS-exposed *Clec2^pltKO^* mice had increased autoantibodies to a wide variety of antigens, including autoantibodies that are typically seen in autoimmune diseases, such as Sm, SSA-A, Jo-1, Scl-70, and autoantibodies to complement proteins (Supplemental Fig. S3, *A–F*) (44, 47, 48).

Because of the close association of TLOs and the lymphatic vasculature, we assessed lymphatic morphology in *Clec2^pltKO^* mice after CS exposure. We found that despite having decreased lymphatic drainage, *Clec2^pltKO^* mice at baseline have an increased density of lymphatic vessel staining in the lung, which likely reflects the abnormal morphology and development of these vessels (Supplemental Fig. S4, *A–D*). Interestingly, *Clec2^pltKO^* mice have detectable lymphatic thrombosis at baseline that is increased with CS exposure (Supplemental Fig. S4, *E–K*), perhaps due to impaired flow and stasis in these vessels and agreeing with our previous studies that have shown that CS promotes prothrombotic lung lymphatics (5, 6).

### Increased Lymphatic Markers Are Associated with Emphysema and Prominent TLOs in Patients with COPD

**Figure 7.**
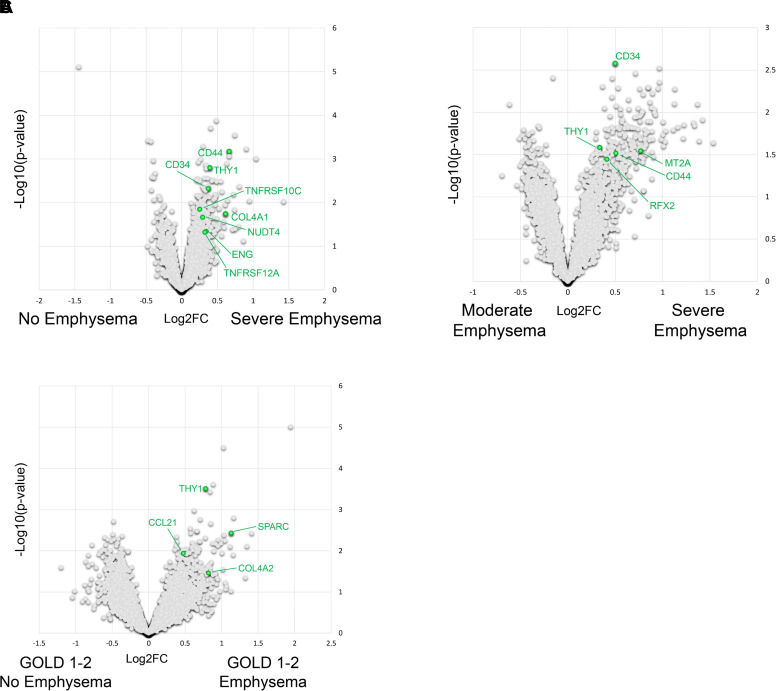
Emphysema phenotype and disease severity are associated with lymphatic markers in patients with COPD compared with patients without emphysema. *A–C*: FFPE lung sections from human patients with COPD and controls were analyzed using Nanostring GeoMX Digital Spatial Profiler. Emphysema was assessed via CT scan and classified according to the %LAA_950_. See Supplemental Table S3 for patient demographics. *A*: volcano plot comparing gene expression in the lung parenchyma of patients with severe emphysema (%LAA_950_ > 17.7) compared with individuals without emphysema (%LAA_950_ < 5.0). *B*: volcano plot comparing gene expression in the lung parenchyma of patients with severe emphysema compared with moderate emphysema (%LAA_950_ 5–17.7). *C*: volcano plot comparing gene expression in the lung parenchyma of patients with GOLD 1-2 patients with COPD with (%LAA_950_ > 5.0) and without emphysema (%LAA_950_ < 5.0). COPD, chronic obstructive pulmonary disease; CT, computed tomography.

Our data in mice indicated a pathogenic role for autoreactive TLOs in mice with lymphatic dysfunction that develop emphysema. We therefore asked whether autoreactive TLOs in human emphysema are characterized by changes in lymphatic markers. COPD is a heterogeneous disease in which diverse endotypes reflect distinct pathological mechanisms of disease. Among these, there is a subset of patients with an emphysema phenotype that is characterized by TLOs, autoantibodies, and self-reactive T-cells that drive lung injury (18, 45, 47–53). We used spatial transcriptomics to analyze lung tissue from patients with COPD that have been identified as having an autoimmune emphysema phenotype and prominent TLOs compared with patients with COPD without emphysema and less prominent TLOs (18). We found increased expression of vascular markers (*Thy1*, *CD34*, and *Col4a*) in the lung parenchyma of patients with severe emphysema compared with patients with COPD without emphysema or with moderate emphysema (Fig. 7, *A* and *B*). We also found increased expression *NUDT4*, which has been recently identified as a marker of lymph node LECs (54) in the lungs of emphysema patients with autoreactive TLOs compared with those with COPD without emphysema (Fig. 7*A*). Interestingly, *Thy1* and *Ccl21* were increased in GOLD 1-2 patients with emphysema and prominent TLOs compared with GOLD 1-2 patients without emphysema (Fig. 7*C*). Though these data are only associative, given that *Ccl21* is expressed almost exclusively by LECs in the human lung (55), these results suggest that lymphatic markers may correlate with an autoimmune emphysema phenotype, even among patients with the same stage of COPD.

## DISCUSSION

Studies in both patients and animal models have shown lymphatic dysfunction and TLO formation in diverse settings of autoimmune and autoinflammatory disease (36–40). Here, we describe an autoimmune phenotype of emphysema due to lymphatic dysfunction in mice that can be partially rescued by blocking TLO formation. Though there are several lines of data suggesting that TLOs are pathogenic in COPD in both humans and animal studies (49, 56–58), defining their importance has been complicated by the fact that lymphoid follicles that form after CS exposure alone are overall dispensable for the development of emphysema in mouse models (32, 58, 59). Though this may be due to the well-described limitations of modeling CS-induced emphysema in mice (60), the finding that CLEC2 KO mice develop severe emphysema within 4 mo, a timepoint that would not be sufficient for this phenotype in wild-type mice, and that blocking TLO formation can partially rescue emphysema in this model suggests a distinct and possibly more pathogenic role for TLOs formed in the setting of lymphatic dysfunction than those formed after CS exposure alone.

A clinical trial of B cell blockade in human COPD was terminated early due to an increased risk of infection (28), calling into question the relevance of TLOs in the pathogenesis of human disease. However, among the clinically heterogeneous population of patients with COPD, pathogenic TLOs are likely only a prominent marker of disease in a subset of patients with an autoimmune emphysema phenotype. Recent data support this concept and have shed light on the clinical and histological signatures of these patients with emphysema and prominent self-reactive TLOs (18, 45, 49). Although we have not uncovered any direct evidence of lymphatic dysfunction playing a causative role in autoreactive TLO formation in human disease, given the association of lymphatic dysfunction with autoimmunity in other settings (38), our data suggest that further investigation is warranted into whether changes in lymphatic function may be present in autoimmune endotypes of COPD.

We have also found that CS exposure results in increased CD8, Batf3, MHCII, and CD40 in preexisting lung TLOs in mice with lymphatic dysfunction. Upregulation of these proteins that may suggest increased T cell memory, antigen presentation, and class-switching in these TLOs after CS exposure. In addition, CS exposure led to increased lung localization of autoantibodies in parallel with increased lymphatic thrombosis in these animals. The mechanism of this change in autoantibody localization is unclear but may reflect alterations in normal mucosal barrier functions with chronic CS exposure. Lymphatic dysfunction is seen in rheumatoid arthritis, Chron’s disease, and SLE, which also have worse outcomes associated with smoking (38, 41–43). Our data support these previous studies and indicate that lymphatic dysfunction can also cause autoimmunity in the lung, perhaps by causing baseline self-reactivity from TLOs that primes the lungs for further injury after CS exposure. This underlying lymphatic dysfunction may be due to genetic or developmental factors, or due to CS itself, which we have shown causes lymphatic dysfunction before the onset of lung injury in mice and is also seen in human emphysema (6). Further studies are warranted to investigate whether CS exacerbates autoimmune disease via a common mechanism by causing further lymphatic damage, by increasing the maturation of self-reactive TLOs formed due to lymphatic dysfunction, or both.

The studies shown here take advantage of lymphatic dysfunction in *Clec2^pltKO^* mice that results from a lack of separation between the blood and lymphatic systems and impaired lymphatic vessel maturation (8, 20, 21). Although these mice also have defects in platelet count and function at baseline, the role of CLEC2 in hemostasis is relatively minor and unlikely to contribute to the phenotype of TLO formation and lung injury that we observe (22, 61, 62). In addition, we and others have shown TLO formation associated with lymphatic dysfunction in other models (4, 24, 40), suggesting that it is lymphatic impairment that drives TLO formation in CLEC2-deficient mice. Interestingly, mice with a lymphatic leukocyte trafficking defect due to loss of CCR7, a cytokine receptor on leukocytes that binds CCL21 on the lymphatic endothelium, also develop TLOs (63) and an autoimmune phenotype (36). In addition, mice lacking the lymphatic transcription factor FOXC2 develop lymphatic dysfunction and lung TLOs (24), with MHC II-expressing LECs. Thus, TLO formation in the lungs may be a common endpoint of lymphatic dysfunction caused by diverse anatomic and molecular mechanisms, with MHC II-expressing LECs a marker of ongoing lymphatic dysfunction and inflammation.

## DATA AVAILABILITY

RNA sequencing data have been deposited in the Gene Expression Omnibus (GEO) database (https://www.ncbi.nlm.nih.gov/geo) under the accession number GSE280059.

## SUPPLEMENTAL MATERIAL

10.6084/m9.figshare.27041914 Supplemental Figs. S1–S4 and Supplemental Tables S1–S4: https://doi.org/10.6084/m9.figshare.27041914.

## GRANTS

This work was supported by K01HL145365 (to H.O.R.), R01HL162990 (to H.O.R.), the Robert Wood Johnson Foundation (to H.O.R.), the Manning Foundation (to H.O.R.), and the American Lung Association Innovation Award AAAALA2023 (to H.O.R.).

## DISCLOSURES

No conflicts of interest, financial or otherwise, are declared by the authors.

## AUTHOR CONTRIBUTIONS

B.S., T.M.L., R.L., and H.O.R. conceived and designed research; B.S., K.K., A.T., T.M.L., J.R.Q., J.C.-C., F.P., R.L., and H.O.R. performed experiments; B.S., K.K., A.T., T.M.L., S.H., J.P.-P., J.R.Q., T.P., F.P., R.L., and H.O.R. analyzed data; B.S., A.T., T.M.L., J.R.Q., R.L., and H.O.R. interpreted results of experiments; T.M.L., J.R.Q., R.L., and H.O.R. prepared figures; H.O.R. drafted manuscript; B.S., A.T., and H.O.R. edited and revised manuscript; B.S., A.T., T.M.L., S.H., J.P.-J., J.R.Q., J.C.-G., T.P., F.P., R.L., and H.O.R. approved final version of manuscript.
